# MASCC/ISOO Clinical Practice Statement: dental evaluation and management prior to treatment for hematologic malignancies and CAR T-cell therapy

**DOI:** 10.1007/s00520-025-09845-4

**Published:** 2025-09-13

**Authors:** Yehuda Zadik, Judith E. Raber-Durlacher, Joel B. Epstein, Alessandra Majorana, Wanessa Miranda-Silva, Sujin Yang, Nicole Blijlevens, Sharon Elad

**Affiliations:** 1 Department of Oral Medicine, and Saligman Clinics, Faculty of Dental Medicine, The Hebrew University of Jerusalem, Hadassah-University Medical Center, P.O. Box 12272, 9112002 Jerusalem, Israel; 2 Department of Oral Medicine, Academic Centre for Dentistry Amsterdam (ACTA), University of Amsterdam and VU University, Amsterdam, The Netherlands; 3 Department of Oral and Maxillofacial Surgery, Amsterdam UMC, University of Amsterdam, Amsterdam, The Netherlands; 4 Cedars-Sinai Medical Center, Los Angeles, CA USA; 5 City of Hope National Cancer Center, Duarte, CA USA; 6 Department of Oral Medicine and Paediatric Dentistry, University of Brescia, Brescia, Italy; 7 Molecular Oncology Center, Instituto de Ensino E Pesquisa, Hospital Sírio-Libanês, São Paulo, Brazil; 8 Department of Advanced General Dentistry, College of Dentistry, Yonsei University, Seoul, Korea; 9 Department of Hematology, Radboud University Medical Center, Radboud Institute for Health Sciences, Nijmegen, The Netherlands; 10 Oral Medicine, Eastman Institute for Oral Health, University of Rochester Medical Center, Rochester, NY USA

**Keywords:** Cancer, CAR-T therapy, Chemotherapy, Dental infection, Geriatric, Hematopoietic cell transplantation, Hemato-oncology, Leukemia, Neutropenia, Pediatric, Prevention

## Abstract

**Purpose:**

A MASCC/ISOO Clinical Practice Statement (CPS) aims to provide clinicians with a guide for managing oral conditions in cancer patients. This CPS addresses dental care prior to chemotherapy, hematopoietic cell transplantation, chimeric antigen receptor (CAR) T-cell therapy, and other treatments for hematologic malignancies in pediatric, adult, and elderly patients. Guidance on optimal timing for resuming dental treatment post-therapy is also included.

**Methods:**

This CPS was developed through a critical evaluation of the literature, followed by a structured discussion of a group of leading experts, members of the Oral Care Study Group of MASCC/ISOO. Information is presented in succinct bullet points and a table to create a practical manual on the best standards of care.

**Results:**

Patients undergoing anti-cancer therapy require a thorough dental assessment and treatment. Pre-therapy dental care aims to prevent oral infection, systemic pathogen spread, oral pain, local irritation, and risk of aspiration and to educate patients on oral hygiene and managing the expected common oral complications. This preventive approach supports oral intake, relieves pain, prevents treatment interruptions, enhances quality of life, and potentially reduces mortality. This CPS outlines the essential considerations for the dental evaluation and treatment, focusing on key dental diagnoses, procedural prioritization, and timing relative to the hemato-oncologic therapy.

**Conclusions:**

This CPS provides clinicians with strategies for effective dental clearance, improving patient outcomes and minimizing complications during and after anti-cancer therapy. CAR T-cell therapy introduces additional considerations in the dental treatment plan before and immediately after the cytoreductive therapy.

**Supplementary Information:**

The online version contains supplementary material available at 10.1007/s00520-025-09845-4.

## Background

Patients diagnosed with hematological malignancies are vulnerable to various local and systemic adverse effects of the hemato-oncologic treatment, mainly pancytopenia-related complications. This is well recognized in myeloablative chemotherapy protocols, including hematopoietic cell transplantation (HCT), and to a lesser extent in non-myeloablative protocols. Likewise, in chimeric antigen receptor (CAR) T-cell therapy, the lymphodepletion phase may induce leukopenia. The main concern is a life-threatening oral infection or hematogenic spread of infection from oral and dental sources. Given that invasive or surgical dental treatments during the hemato-oncologic treatment may be contraindicated, it is vital to eliminate foci of oral infection prior to the initiation of therapy. Moreover, dental morbidities may aggravate oral mucositis (OM), which is associated with intense pain and may impair the patient’s ability to withstand the cancer therapy. Accordingly, it is recommended to have dental evaluation and treatment of pertinent dental needs prior to initiating cancer therapy in hemato-oncologic patients. This procedure is known as *dental clearance*. The value of dental clearance was demonstrated in a decision analysis which identified a higher mortality risk if dental needs are untreated before HCT [1]. This study concluded that an additional 1.8 patients of every 1000 patients will die secondary to dental infection when “no dental treatment” strategy is employed compared to when patients have “dental treatment.” Another study found that dental clearance prior to initiating chemotherapy in patients with acute myeloid leukemia (AML) leads to a significant six-fold reduction in dental emergencies occurring during hospitalization for induction chemotherapy among screened patients compared to unscreened patients [2]. Additionally, a greater number of oral foci of infection before HCT were associated with longer stay in the hospital after HCT [3].

A consensus meeting addressing this topic was held in San Antonio in 2008 [4]. Since then, significant advances in cancer therapy, including the introduction of CAR T-cell therapy, have underscored the need for updated guidelines regarding pretherapy dental evaluation and clearance for patients with hematologic malignancies [5]. Therefore, a working group of the Oral Care Study Group (OCSG) of the Multinational Association of Supportive Care in Cancer and the International Society of Oral Oncology (MASCC/ISOO) composed this expert-opinion Clinical Practice Statement (CPS). In the absence of formal society guidelines, this CPS is the road map to the optimal dental clearance of patients prior to chemotherapy, HCT, CAR T-cell, or other treatment of hematologic cancer.

## Objective

The aim of this CPS is to concisely outline the dental approach recommended before treatment of hematological malignancy, either chemotherapy, HCT, CAR T-cell therapy, or other specific modalities. Additionally, this CPS is aimed to provide indicators about the optimal timing to resume dental treatment following these anti-cancer therapies. Both adult and pediatric patient populations are addressed.

## Methods

A critical evaluation of the literature was conducted by a group of leading experts, members of the OCSG of MASCC/ISOO. PubMed was searched on data pertinent to dental evaluation prior to hemato-oncologic therapy, HCT or CAR T-cell therapy, in the timeframe since inception up to August 1, 2024, for clinical trials assessing the risk for local and systemic infection with and without dental treatment prior to hemato-oncologic therapy. Previous statements by leading organizations and societies were reviewed as part of the consensus development process [4, 6–13]. During the development of the manuscript, point questions that merited a focused assessment were generated, and a specific literature search was done to ensure accuracy of information. The draft was then discussed in a multi-step process by an international working group of the OCSG of MASCC/ISOO and approved by two independent boards: the ISOO Advisory Board and the MASCC Guidelines Committee.

This CPS also applies to patients with solitary hematological tumors treated with radiotherapy (RT) to the head and neck (e.g., lymphoma or plasmacytoma) although if the RT is the single treatment modality, these patients are not typically at risk for systemic immunosuppression. Additionally, this CPS applies to HCT due to diagnoses other than hematologic malignancies, in which the patient undergoes the same process of conditioning regimen-induced immunosuppression. This CPS does not apply to dental treatment or oral complications following chemotherapy, HCT, CAR T-cell therapy, or other anti-cancer therapy for hemato-oncologic malignancies.

## Management

- The identification and management of dental needs prior to initiating hemato-oncologic therapy are prudent for all patients, including those who regularly receive oral health care [14]. The dental evaluation should include a clinical and radiological assessment. Ideally, periodontal assessment should be performed if the medical background and blood counts permit (see below).
- The pre-cancer dental management is determined by the following goals:
  o To eliminate dental sources of infection to the extent of reducing the risk of acute dental infection or reactivation of chronic infection until immune reconstitution occurs and to prevent systemic spread of dental and oral pathogenic micro-organisms during this period.
  o To reduce oral discomfort or pain that may be a burden during cancer treatment.
  o To reduce local irritation from the teeth, dental restorations or prostheses to the oral mucosa while the tissue is at risk for OM.
  o To reduce the risk for aspiration of loose dental structures.
  o To educate the patient about the importance of self-care, oral hygiene during the hemato-oncologic cancer therapy, and briefly prepare the patient for common anticipated oral complications.
  o Ultimately, to improve oral intake, reduce pain, prevent interruption in cancer therapy, improve quality of life, and possibly reduce mortality. Indirectly, this may also reduce costs of care.
- Accordingly, the treatment plan should prioritize dental needs that may develop into active infection, may cause uncontrolled pain or irritation of the oral mucosa during the immunosuppression, or pose a risk for aspiration.
- Examples of dental needs at high priority to be addressed pre hemato-oncologic therapy include symptomatic teeth, advanced dental caries approaching the pulp, unrestorable teeth, inflamed or necrotic pulp, symptomatic apical periodontitis, symptomatic apical abscess, gingival or periodontal abscess, advanced chronic periodontitis with purulent exudate or with severe tooth mobility, periimplantitis with purulent exudate or with severe implant mobility, pericoronitis during the last three months, loose contact point with food impaction, a sharp structure (e.g., fractured tooth or restoration and ill-fitting prosthesis or orthodontic appliances) that are traumatic to the soft tissue, or pathologies that typically deem an immediate excision. A “symptomatic” dental need denotes symptoms occurring within the last three months.
- Poor oral hygiene (OH) is an aggravating factor for oral infection that drives more determinative dental treatment decisions. OH instruction (OHI) and professional OH treatment are key elements in the dental treatment plan that will help to set a healthy plaque-control at baseline and educate the patient on how to maintain it. Given that fatigue is a common complication following hemato-oncologic treatments, particularly CAR T-cell therapy, as part of their education, patients should be encouraged to maintain good OH despite this challenge.
- There is no reservation regarding continuing tooth brushing [15]. Inter-dental brushing/flossing should be continued during the HCT or cytopenia period if a patient is proficient with these techniques. The toothbrush should be rinsed thoroughly with water and allowed to air-dry and should be replaced at the first signs of bristle wear [15]. Removable dentures or appliances should be cleaned daily mechanically and with agents specifically designated for this purpose. Chlorhexidine (CHX) rinse may be an effective adjunct in plaque control. Alcohol-free CHX may be used if the oral mucosa is sensitive to standard CHX preparation.
- Fluoride application, either office-based or home-based, may be offered as a preventive measure in dentate patients [9].
- The time constraints prior to initiation of cancer therapy dictate modifications to the dental treatment plan. The burden of the dental disease, the urgency of the cancer treatment, and the risk of complications due to the dental treatment are the main considerations in prioritizing the dental treatment before the initiation of the cancer therapy. Insufficient time for post-operative healing or for re-treatment if the first attempt fails drives a more determinative approach over a conventional dental treatment plan in healthy patients. Multi-visit, time-consuming endodontic treatment is unfavored, especially when additional high-priority dental needs are present.
- The dental treatment plan should address present dental needs as well as dental conditions that are anticipated to deteriorate within 6–12 months following the hemato-oncologic therapy, until routine dental treatment may be resumed (see below the section about the considerations to restart routine dental care).
- Considering the time constraints, efforts should be made to consolidate multiple treatments into a single session to efficiently manage the limited timeframe available. It is advised to schedule dental extractions as early as possible to maximize the postoperative healing period before the initiation of the hemato-oncologic therapy. Ideally, tooth extractions should be performed with sufficient time before hemato-oncologic therapy to allow the socket to heal with epithelium, thereby reducing the risk of pathogen penetration. This is estimated to happen within 2–3 weeks following the extraction.
- The decision about tooth extraction is a delicate balance, and the dentist should be mindful of its impact. Post-extraction adverse sequelae (e.g., infection and dry socket) may potentially postpone the oncologic treatment. Additionally, a radical treatment plan may negatively impact oral function in the immediate term. Furthermore, the patient may have compromised oral function for a long term because routine dental care is held.
- Ideally, if time allows, a dental treatment plan should extend beyond mere tooth extraction to include restorative treatment, which may potentially reduce the risk of bacteremia and would improve the patient’s quality of life [16].
- If time constraints do not allow delivering all planned dental care, the patient should be informed about unresolved dental needs posing a low risk of immediate complications. These dental needs should be addressed following the hemato-oncologic therapy when the medical condition permits, for example during the next remission phase. For unresolved incipient dental caries, an in-office fluoridation and at-home high-concentration fluoride toothpaste may be offered. There may be geographic variation in the availability of these fluoride agents.
- The hematologist should be informed about the dental needs which pose a high risk of complications and should be addressed in the immediate term. Communication about the invasiveness of the planned treatment and expected healing time is of great value in order for the physician to advise on the delivery of the dental treatment or to postpone the chemotherapy, HCT, or CAR T-cell therapy.
- When dental treatment is deemed necessary prior to the hemato-oncologic therapy, adjustment of the treatment plan may be needed based on the patient’s hematologic profile and medical status.
- *Risk of spread of oral pathogenic micro-organisms during dental treatment*. Clinician discretion regarding antibiotic prophylaxis in dental patients at risk for infection is needed (Table 1).
- *Risk of bleeding*. In thrombocytopenic patients, major bleeding may develop following surgical or non-surgical intervention, such as scaling (Table). In addition, cancer patients are at risk of coagulation disorders, and some may receive anti-coagulant treatment. The clinician should assess the bleeding risk and prepare for intra-operative and post-operative bleeding complications.
- *Risk of anemia-related complications*. Patients with anemia may experience fatigue and dizziness, especially upon position change (Table). Additionally, anemia may also delay healing.
- *Approach to indwelling catheter*. Often patients prior to hemato-oncologic therapy undergo indwelling catheter insertion. Antibiotic prophylaxis is not indicated prior to dental intervention in immunocompetent patients with indwelling catheter [17].
  o *Risk of osteoradionecrosis (ORN)*. The radiotherapy dose in patients with orofacial lymphoma or plasmacytoma is relatively low. However, if the cumulative dose to the jawbone at the site of extraction exceeds 44 Gy, ORN may develop [18]. Thus, the treatment approach for these hematologic patients will be similar to patients with head and neck cancer. It is advised to assess if extraction is needed prior to the radiotherapy and complete the extractions > 2 weeks prior to the radiotherapy. Dosimetry mapping may assist in determining the risk of developing ORN in a particular jaw segment, and accordingly in the planning of dental treatment.
  o *Comorbidities*. The patient’s medical records may identify medical comorbidities and medications that require additional modifications in the dental treatment plan. For example, history of treatment with bone modifying agents (e.g., bisphosphonates and denosumab) poses a higher risk for medication-related osteonecrosis of the jaw (MRONJ), and therefore, the dental treatment plan should be adjusted according to standard recommendations [19].
- Given the complexity of the decision-making in the dental management of hemato-oncology patients prior to the cancer therapy, a consultation with an oral medicine specialist or a hospital dentist with expertise in managing this patient population is advised.
- The dental practitioner should educate the patient on managing the common oral complications associated with upcoming hemato-oncologic therapy [20]. Detailed information on these complications is outside the scope of this CPS paper; clinicians are referred to other MASCC/ISOO publications for guidance on managing these issues (see Suggested Reading as Electronic Supplementary Material).
- Routine dental treatment may be resumed after immune reconstitution.
- In hemato-oncologic patients treated with chemotherapy, bone marrow recovery is expected within about 2–3 weeks following the last dose of chemotherapy, as can be indicated by the white blood cell (WBC) count, and particularly neutrophil counts.
- In patients undergoing HCT, the immune reconstitution status is usually substantiated by the WBC, CD4 count, and immunoglobulin levels, among other tests. Often, this is attained at about 3 months post autologous HCT and about 6 months post allogeneic HCT. The transplant physician may choose to postpone the dental intervention beyond this period to assure the patient’s immunocompetence and ability to undergo dental treatment safely.
- In the setting of CAR T-cell therapy, cytopenia may develop later and last longer than post conventional HCT [21]. Blood counts should be tested prior to invasive dental treatment during the first 2 years following the CAR T-cell therapy. Given the unpredictable risk for cytopenia, the patient should be motivated to practice good oral hygiene especially while routine dental treatment cannot be performed.
- If an acute dentoalveolar infection develops prior to immune reconstitution, nonsurgical intervention including systemic antibiotics and local antimicrobials are the mainstay of treatment. Intervention for dental complications during cancer therapy and prior to immune reconstitution is reserved for exceptional cases and requires a consultation between the dental care provider and the hematologist.
- Following CAR T-cell therapy, if a dental emergency develops while the patient suffers prolonged cytopenia, and topical antimicrobial and systemic antibiotic treatment is ineffective in controlling the infection, dental treatment may be delivered as a last resort. In such cases, administration of granulocyte colony-stimulating factor (G-CSF) may be considered to support dental interventions, provided that the patient does not have a concurrent diagnosis of cytokine release syndrome [21].
- Preparatory treatment with G-CSF may be considered on an individual basis in other neutropenic patients in need of dental intervention, for example, patients post HCT with delayed engraftment. When G-CSF treatment is considered, the hematologist should be consulted.
- In pediatric patients, there are a few unique considerations prior to chemotherapy or HCT in addition to the removal of foci of infection:
- Oral hygiene aids should be adjusted to the patient’s age. The parents should be actively involved in the daily oral hygiene care. In younger or critically ill pediatric patients who are unable to brush teeth or use mouth rinses, the dental plaque can be mechanically removed by gently massaging the gumline with a gauze soaked with saline or CHX.
- Primary teeth should be allowed to exfoliate naturally. However, extremely mobile teeth should be removed as part of the dental clearance. The rationale is to reduce the risk for pain, ensure oral intake, and avoid potential bleeding during thrombocytopenia.
- Given the time constraints and behavioral challenges in pediatric patients, silver diamine fluoride (SDF) may be considered a non-restorative treatment to arrest superficial carious lesions on primary teeth [22]. Patients and their parents should be informed about the possible side effects of SDF treatment and that future restoration will be needed to restore the cavitated tooth or that SDF treatment may need to be repeated periodically.
- The treatment delivery should be adjusted to the pediatric patient population in order to achieve the patient’s collaboration. For example, behavioral-cognitive techniques, sedation, or involvement of supporting services (medical clowns, animal-therapy). When there is an abundance of dental needs, general anesthesia may be required. Pediatric dental specialists are well experienced and equipped to manage this patient population.
- Orthodontic brackets and appliances that may be plaque-retentive or traumatic to the soft tissues should be removed.
- In elderly patients, there are a few unique considerations prior to chemotherapy or HCT in addition to the removal of foci of infection:
- Removable dentures and implant-based prostheses are common in elderly patients.
- In removable denture users, denture-induced traumatic contacts on a mucosal-bearing area should be removed prior to cancer therapy. Relining may be needed to fix ill-fitting/unstable dentures.
- Patient should be educated about the optimal care for the denture. Briefly, regular rinses of the dentures in water are recommended. Additionally, periodic decontamination of the dentures with sodium bicarbonate, CHX, or other commercial products designated for denture cleaning is advised. Likewise, the patient should be educated about the adequate use of the denture specifically the removal of the denture at night.
- In patients with implant-based fixed prostheses, the importance of self-oral hygiene adjacent to the dental implants should be stressed, and adjusted dental hygiene aids should be recommended.

**Table 1:** Considerations for dental management of cancer patients with abnormal blood counts

Risk	Clinical implications and treatment modifications
Spread of oral pathogenic micro-organisms during dental treatment	• In severe neutropenia (neutrophil count < 500 cells per microliter), only dental treatment that is a medical necessity is advised • If invasive dental treatment is deemed necessary, antibiotic prophylaxis may be suggested for neutropenic patients to prevent bacteremia, and particularly in the case of symptomatic dental infection. Clinician discretion is essential—the more severe the neutropenia and the more invasive the dental procedure, the stronger the consideration for antibiotic prophylaxis • It is challenging to select the antibiotics, as in patients with hematologic cancer who receive multiple chemotherapy cycles or admitted to hospital there may have been a shift in the oral flora • The antibiotic is administered 30–60 minutes prior to the invasive dental procedure. The commonly prescribed antibiotics protocols include ○ Amoxicillin 2 g ○ Doxycycline 100 mg ○ Cephalexin 2 g ○ Azithromycin or clarithromycin 500 mg • The principles underlying these protocols are described in the literature [23, 24] Likewise it details protocols for patients unable to take oral medications, and intravenous or intramuscular antibiotics may be used • The decision about antibiotic prophylaxis should be made collaboratively with the dentist, dental oncologist, and hemato-oncologist, considering the patient’s oncologic condition, history of infections, presence of symptomatic dental infection, complexity of dental procedure, and unique center-related considerations (e.g., if data for oral flora survey available) • The patient should be informed about the risk and benefit of the antibiotic prophylaxis • The antibiotic regimen should be alternated if the interval between sequential invasive dental appointments is < 10 days • In neutropenic patients, probing through an infected site should be considered carefully to avoid spread of the infection, and if needed, it may be grouped with a dental treatment for which antibiotic prophylaxis is prescribed • During and following an invasive procedure, actions to reduce infection are advised, such as pre-operative chlorhexidine rinses and post-operative copious saline irrigations • In hemato-oncology patients the WBC count may not represent the actual number of functional WBCs, and a consult with the hematologist about the immune status may be needed • Suggested primary closure if possible and no intra-alveolar materials placed • If extraction is needed, primary closure without placement of intra-alveolar materials is preferable. There is no evidence in the literature about the safety of bone graft insertion prior to chemotherapy, HCT or CAR T-cell therapy, and therefore the risk associated with alveolar ridge preservation is unknown
Bleeding	• To control bleeding in invasive procedures local pressure and/or sutures should be applied. Topical hemostatic agents are an important adjunct • If intra-socket hemostatic agents are considered, rapidly resorbable agents are preferred over long-standing hemostatic agents as the latter may delay healing or may be a nidus for infection • In cases of extremely low platelet count, the care provider should weigh the benefits and risks of the dental treatment. Any surgery must be planned and coordinated with the hemato-oncologist to assess risk versus benefit • Platelet or fresh frozen plasma transfusion may be needed prior and subsequent to the dental procedure, and if so, the physician should be consulted • If platelet refractoriness is diagnosed, complete blood count should be tested following the platelet transfusion and prior to the dental treatment to confirm sufficient platelet count at baseline • Coagulation testing and hemostatic measures may be required before and during dental interventions, respectively, in patients undergoing anticoagulant therapy
Anemia-related complications	• In anemic patients, the compensation for posture-changes or stressful situations may be compromised, in particular if the hemoglobin drop is rapid. The dentist should monitor vitals and take measures to prevent falls • Healing may be delayed in anemic patients. Therefore, surgical techniques should facilitate primary closure, and patient should be followed up to confirm healing • In cases of severe anemia, caution should be exercised during dental procedures performed under general anesthesia or sedation, ensuring the administration of at least 50% oxygen concentration • Any surgery must be coordinated with the hemato-oncologist to assess risk versus benefit. Erythropoiesis-stimulating agent may be considered [25]

*CAR* chimeric antigen receptor, *HCT* hematopoietic cell transplantation, *WBC* white blood cell

In summary, dental clearance serves multiple purposes and involves numerous considerations requiring a comprehensive understanding of the patient’s dental needs and the nature and urgency of the cancer therapy. The decision should be individualized, taking into account patient-specific factors, cancer characteristics, planned cancer treatment, and dental-oral diagnosis. Achieving optimal outcomes is best facilitated in a multidisciplinary setting with experienced healthcare providers.

## Supplementary Information

Below is the link to the electronic supplementary material.

Supplementary file1 (DOCX 19.6 KB)

## Data Availability

No datasets were generated or analysed during the current study.

## Declarations

### Disclaimer

The MASCC/ISOO OCSG Statements have been developed to facilitate the expert-opinion based management of oral complications of cancer therapy where high-quality evidence is lacking. Clinicians should use their judgment when making treatment decisions for individual patients. The Statement authors and the MASCC/ISOO do not guarantee or take responsibility for clinical outcomes in individual patients.
