# Supplementary material for: MASCC/ISOO Clinical Practice Statement: dental evaluation and management prior to treatment for hematologic malignancies and CAR T-cell therapy
Source: Support Care Cancer. 2025 Sep 13;33(10):853. doi: 10.1007/s00520-025-09845-4 (PMC12433334; doi:10.1007/s00520-025-09845-4)
Supplement: Supplementary file 1 — Supplementary file1 (DOCX 19.6 KB) [file 520_2025_9845_MOESM1_ESM.docx]

**MASCC/ISOO Clinical Practice Statement: dental evaluation and management prior to treatment for hematologic malignancies and CAR T‑cell therapy**

**Suggested reading**

1. Elad S, Zadik Y, Yarom N (2017) Oral complications of nonsurgical cancer therapies. Atlas Oral Maxillofac Surg Clin North Am. 25(2):133-147. doi: 10.1016/j.cxom.2017.04.006. PMID: 28778303.

2. Schuurhuis JM, Span LF, Stokman MA, van Winkelhoff AJ, Vissink A, Spijkervet FK (2016) Effect of leaving chronic oral foci untreated on infectious complications during intensive chemotherapy. Br J Cancer. 114(9):972-8. doi: 10.1038/bjc.2016.60. PMID: 27002936; PMCID: PMC4984907.

3. Usmani S, Choquette L, Bona R, Feinn R, Shahid Z, Lalla RV. Transient bacteremia induced by dental cleaning is not associated with infection of central venous catheters in patients with cancer. Oral Surg Oral Med Oral Pathol Oral Radiol. 125(4):286-294. doi: 10.1016/j.oooo.2017.12.022. Epub 2018 Jan 11. PMID: 29428697; PMCID: PMC5944361.

4. Yarom N, Shapiro CL, Peterson DE, Van Poznak CH, Bohlke K, Ruggiero SL, et al (2019) Medication-related osteonecrosis of the jaw: MASCC/ISOO/ASCO Clinical Practice Guideline. J Clin Oncol. 37(25):2270-90. doi: 10.1200/JCO.19.01186. PMID: 31329513.

5. Elad S, Cheng KKF, Lalla RV, Yarom N, Hong C, Logan RM, et al; Mucositis Guidelines Leadership Group of the Multinational Association of Supportive Care in Cancer and International Society of Oral Oncology (MASCC/ISOO) (2020) MASCC/ISOO clinical practice guidelines for the management of mucositis secondary to cancer therapy. Cancer. 126(19):4423-31. doi: 10.1002/cncr.33100. doi: 10.1002/cncr.33549. PMID: 32786044; PMCID: PMC7540329.

6. Haverman TM, Raber-Durlacher JE, Raghoebar II, Rademacher WMH, Rozema FR, Hazenberg MD, et al (2020) Oral chronic graft-versus-host disease: What the general dental practitioner needs to know. J Am Dent Assoc. 151(11):846-856. doi: 10.1016/j.adaj.2020.08.001. PMID: 33121606.

7. Elad S, Aljitawi O, Zadik Y (2021) Oral graft-versus-host disease: A pictorial review and a guide for dental practitioners. Int Dent J. 2021 Feb;71(1):9-20. doi: 10.1111/idj.12584. PMID: 33616057; PMCID: PMC9275209.

8. Hansen HJ, Estilo C, Owosho A, Solano AK, Randazzo J, Huryn J, et al (2021) Dental status and risk of odontogenic complication in patients undergoing hematopoietic stem cell transplant. Support Care Cancer. 29(4):2231-2238. doi: 10.1007/s00520-020-05733-1. PMID: 32901321; PMCID: PMC8432266.

9. Correa MEP, Granzotto FCN, Innocentini LMAR, Reis TC, de Lima EM, Varanda RF, et al (2023) Brazilian dental consensus on dental management in hematopoietic stem cell transplantation, Part I: pre-HSCT. Hematol Transfus Cell Ther. 45(3):358-67. doi: 10.1016/j.htct.2023.04.001. PMID: 37295969; PMCID: PMC10499583.

10. Mirfendereski P, France K (2023) patient receiving hematopoietic stem cell transplantation reports for dental clearance. Dent Clin North Am. 67(3):447-451. doi: 10.1016/j.cden.2023.02.016.

11. Elad S, Yarom N, Zadik Y (2023) Immunotherapy-Related Oral Adverse Effects: Immediate Sequelae, Chronicity and Secondary Cancer. Cancers (Basel). 15(19):4781. doi: 10.3390/cancers15194781. PMID: 37835475; PMCID: PMC10571987.

12. Dean D, Lee SJ, Cutler C, Gooley TA, Hujoel P, Oh UY, et al (2024) Dental evaluation and clearance prior to allogeneic hematopoietic cell transplantation. Oral Dis. 30(4):2635-2644. doi: 10.1111/odi.14717. PMID: 37650229; PMCID: PMC10902180.

13. Hong C, Jensen SB, Vissink A, Bonomo P, Santos-Silva AR, Gueiros LA, et al (2024) MASCC/ISOO Clinical Practice Statement: Management of salivary gland hypofunction and xerostomia in cancer patients. Support Care Cancer. 32(8):548. doi: 10.1007/s00520-024-08688-9. PMID: 39048728.

14. Raber-Durlacher JE, Treister NS, Zadik Y, Dean DR, Miranda-Silva W, Fregnani ER, et al (2024) MASCC/ISOO Clinical Practice Statement: The risk of secondary oral cancer following hematopoietic cell transplantation. Support Care Cancer. 32(8):545. doi: 10.1007/s00520-024-08685-y. PMID: 39048762; PMCID: PMC11269467.

15. Villa A, Epstein JB, Yarom N, Hong C, Fulop C, Bossi P, et al (2024) MASCC/ISOO Clinical Practice Statement: Management of oral complications of targeted therapy. Support Care Cancer. 32(8):549. doi: 10.1007/s00520-024-08689-8. PMID: 39048808.

16. Nair AH, Patel T, Nair AR, Krishnan NA, Balasubramanian D, Iyer S, et al (2024) Oral management of patients undergoing head and neck cancer treatment. J Maxillofac Oral Surg. 23(4):783-792. doi: 10.1007/s12663-023-01911-w. PMID: 39118922; PMCID: PMC11303626.

17. Al-Ansari S, Stolze J, Bresters D, Brook AH, Laheij AMGA, Brand HS, et al (2024) Late complications in long-term childhood cancer survivors: What the oral health professional needs to know. Dent J (Basel). 12(1):17. doi: 10.3390/dj12010017. PMID: 38275678; PMCID: PMC10813876.

18. Abdolrahmani A, Epstein JB, Samim F (2024) Medication-related osteonecrosis of the jaw: evolving research for multimodality medical management. Support Care Cancer. 32(4):212. doi: 10.1007/s00520-024-08388-4. PMID: 38443685.

19. Zadik Y, Raber-Durlacher JE, Epstein JB, Majorana A, Laheij A, Bardellini E, et al (2024) MASCC/ISOO Clinical Practice Statement: Management of oral manifestations of chronic graft-versus-host-disease. Support Care Cancer. 32(8):546. doi: 10.1007/s00520-024-08686-x. PMID: 39048807; PMCID: PMC11269426.
